# A Multiplex LC-MS/MS Approach for Quantifying Antiepileptic Drugs for Therapeutic Drug Monitoring: Validation of 15 Representative Tests

**DOI:** 10.21627/hs00ns84

**Published:** 2026-08-26

**Authors:** Xiaoying Tang, Ruhan Wei, Emily Chegwidden, Richard Giles, Adam M. Kutnick, Drew Payto, Jessica M. Colón-Franco

**Affiliations:** Department of Laboratory Medicine, Cleveland Clinic Foundation, Cleveland, OH, United States; Department of Laboratory Medicine, Cleveland Clinic Foundation, Cleveland, OH, United States; Department of Pathology, Duke University School of Medicine, Durham, NC, United States; Department of Laboratory Medicine, Cleveland Clinic Foundation, Cleveland, OH, United States; Department of Laboratory Medicine, Cleveland Clinic Foundation, Cleveland, OH, United States; Department of Laboratory Medicine, Cleveland Clinic Foundation, Cleveland, OH, United States; Department of Laboratory Medicine, Cleveland Clinic Foundation, Cleveland, OH, United States; Department of Laboratory Medicine, Cleveland Clinic Foundation, Cleveland, OH, United States

**Keywords:** antiepileptic drug, therapeutic drug monitoring, LC-MS/MS, clinical laboratory, method validation

## Abstract

INTRODUCTION: Therapeutic drug monitoring of antiepileptic drugs is critical for optimizing clinical outcomes, minimizing toxicity, assessing drug compliance, and managing overdoses and drug interactions. OBJECTIVES: We developed and validated an LC-MS/MS method for the quantification of 15 antiepileptic drugs (ethosuximide, primidone, pentobarbital, carbamazepine-10,11 epoxide, pregabalin, gabapentin, zonisamide, lacosamide, rufinamide, felbamate, lamotrigine, topiramate, 10,11-dihydro-10-hydroxycarbamazepine, perampanel, and brivaracetam). METHODS: Antiepileptic drugs were extracted from plasma and serum using methanol-based protein precipitation, followed by dilution. A commercial ClinCal® 3-point calibrator was used for all analytes except pentobarbital, which used a 6-point in-house calibrator. Chromatographic separation was achieved using a reverse-phase C18 column with a 7.31 min elution gradient of water and methanol, both containing 2 mM of ammonium acetate. RESULTS: All analytes required a 5 µL injection volume, except ethosuximide and pentobarbital, which required 20 µL and 10 µL, respectively, for optimal performance. The method demonstrated excellent linearity for all 15 antiepileptic drugs within their respective concentration ranges, with acceptable selectivity, accuracy (80.0–118.3%), and intraday and interassay precision (CV < 7.4%). Stability studies showed that antiepileptic drugs were stable in serum and plasma for up to 24 h at room temperature, 7 days at 4 °C, and 30 days at −20 °C, and after extraction for 7 days at 4 °C. CONCLUSION: This LC-MS/MS method supports routine quantification of 15 antiepileptic drugs in a clinical laboratory using a common sample preparation and chromatography workflow across all measurements. It was operationalized into three instrument methods to accommodate the different injection volume and calibrator requirements.

## Non-Specialist Summary

This work describes a fast and easy laboratory method for measuring the concentration of medications used to treat epilepsy using an LC-MS/MS instrument. It was validated for 15 different drugs, including newer medications such as lacosamide, brivaracetam, and perampanel. The method offers flexibility because it uses simple standardized sample preparation and a three-point calibration set that can be used to measure up to 28 different antiepileptic medications.

## 1. Introduction

In the United States, nearly 2.9 million adults have active epilepsy, of whom approximately 85% are taking antiseizure medications, commonly referred to as antiepileptic drugs (AEDs) [1]. Additionally, the 2022–2023 National Survey of Children’s Health estimated that 432,651 children aged under 17 years have epilepsy or a seizure disorder [2]. To manage epilepsy pharmacologically, patients and clinicians have access to more than 30 primary AEDs available for prescription as of 2024 [3]. Therapeutic drug monitoring (TDM) of AEDs is considered the standard of care to optimize treatment, as these drugs may exhibit dose-dependent kinetics, drug-drug interactions, and narrow therapeutic ranges [4,5]. Multiple factors modulate the relationship between the prescribed dose and the drug’s clinical effect. TDM is particularly useful during dose optimization, formulation changes, identification of noncompliance, uncontrolled seizures, suspected toxicity, and altered pharmacokinetics (e.g., during pregnancy, liver or kidney disease, infections, drug-drug interactions, or when pharmacogenetic mutations are present). Depending on the specific drug, AEDs may be approved for monotherapy and/or adjunctive therapy across various seizure types and indications. AEDs are typically classified as first-, second-, and third-generation, depending on their development or introduction date to the market [6].

In clinical laboratories, AED testing is performed using various measurement methods, primarily liquid chromatography-tandem mass spectrometry (LC-MS/MS) and immunoassay (IA), while a minority of laboratories continue to use high-pressure liquid chromatography (HPLC) (and laboratory proficiency testing reports [7]). Factors to consider when deciding which method to use include test availability and regulatory framework, such as whether the test is available as an in vitro diagnostic test approved by the U.S. Food and Drug Administration (FDA) or as a laboratory-developed test. Additional considerations include test throughput, cost-effectiveness, turnaround time (TAT), sensitivity, and selectivity. In IA methods, the quantification of an analyte relies on the formation of an immune complex between the analyte (i.e., antigen) and a highly specific antibody. There is a wide range of IA formats for TDM applications with varying detection methods, coupling chemicals for antigens and antibodies and sensitivities, many of which have been automated [8]. These are commonly available in automated analyzers, making this a desirable option for high-volume testing and tests with rapid TAT requirements. IAs are limited to single-analyte detection and are susceptible to cross-reactivity with related or unrelated compounds [3]. For example, interferences have been reported for carbamazepine from its active metabolite carbamazepine-10,11 epoxide (CARBEP), with assay-dependent cross-reactivity reported ranging from negligible to greater than 90% [9]. Recently, reports indicated that brivaracetam (BRIV) is cross-reactive with levetiracetam IAs [10]. In patients being monitored for more than one AED, this limitation increases sample requirements and cost.

HPLC methods with ultraviolet (UV) detection have been used for TDM in clinical laboratories because of their high reliability, low cost, and ease of use, although their use has become less widespread with the emergence of newer technologies. LC-MS/MS methods have become important analytical tools for AED quantification due to their superior specificity and ability to measure several drugs and metabolites in a single sample and analysis [7]. However, LC-MS/MS techniques are often associated with high initial capital costs and method development requirements [11]. As MS has become more widely adopted in clinical laboratories, its cost-effectiveness and analytical advantages have become increasingly evident, along with the potential for long-term cost savings. Access to LC-MS/MS has also improved: Modern instruments are more rugged, workflows are more streamlined, and usability has increased substantially. These gains are supported by the expanding availability of commercial reagents and tools, including calibrators, standards, data-analysis software, and automation solutions, as well as a growing body of published methods that facilitate implementation [11].

AEDs are classified into three generations based on their time of approval for clinical use [5]. The first-generation AEDs were commonly used until the mid-1990s as first-line treatments but are associated with more severe side effects and drug-drug interactions. A second generation of more effective and less toxic AEDs was subsequently introduced. The third-generation AEDs include drugs approved in the 21st century and are characterized by more specific mechanisms of action that support individualized therapy. We present a method for the simultaneous quantification of first-generation AEDs (i.e., ethosuximide [ETHOS], primidone [PRIM], pentobarbital [PENTOB], and carbamazepine and its active metabolite), as well as second- and third-generation AEDs (i.e., pregabalin [PBALIN], gabapentin [GABA], zonisamide [ZONIS], lacosamide [LACOS], rufinamide [RUFIN], felbamate [FELBA], lamotrigine [LMTR], topiramate [TOPIR], oxcarbazepine [OXCARB] and its metabolite (±)-10,11-dihydro-10-hydroxycarbamazepine [10HYDROCARB], perampanel [PRMP], and BRIV) in a clinical laboratory setting. This method utilizes a streamlined sample preparation process and a single analytical method to quantify multiple AEDs present in a convenient commercial 3-point calibrator product. Additionally, the method presented here can be adapted, using a separate calibrator, for the quantification of PENTOB, a barbiturate used for managing status epilepticus and used off-label to induce medical coma and reduce intracranial pressure in patients with severe brain injury.

## 2. Materials and Methods

The assay method is schematically depicted in Figure 1 and detailed below.

**Figure 1.**
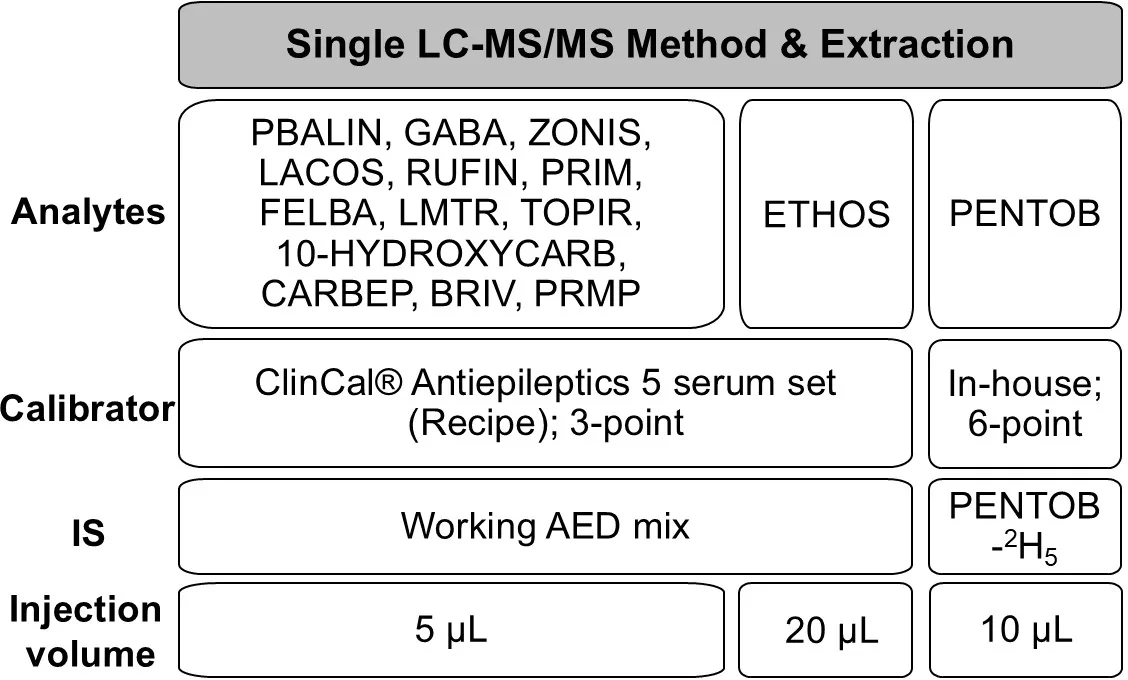
Schematic representation of the assay method.

### 2.1. Reference Materials and Internal Standards

Hereafter, the analytes in this method are referred to by the abbreviations provided below, and collectively, as AEDs. Certified reference materials for CARBEP, FELBA, LMTR, 10HYDROCARB, PENTOB, ZONIS, PBALIN, GABA, LACOS, RUFIN, PRIM, TOPIR, OXCARB, phenobarbital (PHENOB), and amobarbital (AMOB) were purchased from Cerilliant (Round Rock, TX, United States). Certified reference materials for BRIV and ETHOS were purchased from Toronto Research Chemicals (North York, ON, Canada). A certified reference standard for PRMP was purchased from Cayman Chemical (Ann Arbor, MI, United States).

Quantification was pursued for 15 drugs (CARBEP, FELBA, LMTR, 10HYDROCARB, PENTOB, ZONIS, PBALIN, GABA, LACOS, RUFIN, PRIM, TOPIR, BRIV, ETHOS, and PRMP). OXCARB is not routinely measured due to its short half-life. It is isobaric with CARBEP, requiring chromatographic separation. We did not quantify PHENOB as it is routinely measured by IA in our laboratory. However, we demonstrate chromatographic separation and the feasibility of measuring it in this panel, if desired. Isotopically labeled IS solutions were purchased from Cerilliant: PBALIN-^13^C_3_ (0.5 µg/mL), GABA-^13^C_3_ (0.5 µg/mL), ZONIS-^13^C_6_ (1.5 µg/mL), LACOS-^13^C,^2^H_3_ (0.5 µg/mL), PRIM-^2^H_5_ (1.0 µg/mL), LMTR-^13^C,^15^N_4_ (1.0 µg/mL), TOPIR-^2^H_12_ (0.8 µg/mL), 10HYDROCARB-^13^C_6_ (0.5 µg/mL), BRIV-^2^H_3_ (0.3 µg/mL), CARBEP-^13^C_6_ and PENTOB-^2^H_5_ (0.8 µg/mL), and Toronto Research Chemicals: FELBA-^2^H_4_ (0.8 µg/mL), ETHOS-^2^H_3_ (20.0 µg/mL), and RUFIN-^15^N,^2^H_2_ (0.5 µg/mL). Two working standard solutions were prepared in LC-MS–grade methanol containing 0.5% acetic acid (Fisher Scientific, Waltham, MA, United States), one for PENTOB-^2^H_5_ and another for the remaining IS solutions, at the concentrations listed above. At the time of this study, there was no viable option for a stable-labeled PRMP IS, and we evaluated an alternate IS, LACOS-^13^C,^2^H_3_, for quantification [8].

### 2.2. Calibrators and Quality Control

The assay used ClinCal® Antiepileptics 5 Serum Calibrator and ClinChek® Antiepileptics 5 Serum Control (RECIPE, Munich, Germany). The set consisted of 3-point calibrators and two levels of quality control (QC) for 28 AEDs and metabolites. Supplementary Table S1 lists the calibrator concentrations of the analytes included in our method. We prepared a mid-QC level by mixing the QC materials. The calibrator and QC did not include PENTOB, for which a 6-point calibration curve with concentrations 3.13, 6.25, 12.50, 25.00, 50.00, and 100.00 µg/mL and two levels of QC were prepared from standard solution in methanol spiked into Lyphochek drug-free serum (BioRad, Hercules, CA, United States). The calibration curves used a weighting factor of 1/x^2^.

### 2.3. Sample Preparation

This study used 327 de-identified residual serum and lithium-heparin plasma patient specimens collected in gel-free collection tubes. Of these, 283 contained endogenous drug levels, and 44 were blank patient serum or plasma used for spiking experiments. This study was approved by Cleveland Clinic’s Institutional Review Board (10–297). A volume of 50 µL of calibrator, the QC or plasma/serum sample, and 100 µL IS solution in a 1.5 mL polypropylene microcentrifuge tube were vortex mixed for 1 min and centrifuged at 16,000 × g for 10 min at room temperature. Supernatant was transferred to a 96-well plate (Analytical Sales & Services, Flanders, NJ, United States), diluted 1:20 with ultrapure deionized water, covered with a cap mat, and vortex mixed for 30 sec. The injection volumes were optimized during method development for optimal signal and chromatography as follows: 20 µL for ETHOS, 10 µL for PENTOB, and 5 µL for all other analytes. We opted to maintain a standardized sample preparation and customize the injection volumes. The autosampler was kept at 4 °C.

### 2.4. Chromatography and Mass Spectrometry Conditions

The chromatographic separation was performed using a Transcend II TLX-2 UHPLC system (Thermo Fisher Scientific, Waltham, MA, United States) with a Thermo Accucore™ C18 column (50 × 2.1 mm, 2.6 µm) and a guard column (Accucore™ C18, 10 × 2.1 mm, 2.6 µm) at ambient temperature. An elution gradient of water (mobile phase A [MPA]) and methanol (mobile phase B [MPB]) was applied, both containing 2 mM ammonium acetate. The gradient started with 97% MPA and decreased linearly to 42% in 4.00 min, held for 0.70 min, stepped down to 5%, and held for 0.60 min. This was followed by a 2.00 min re-equilibration. The flow rate was 0.6 mL/min. The total assay run time was 7.31 min with data collection starting at 0.70 min for 4.22 min.

Mass analysis was performed on a TSQ Quantis™ triple quadrupole mass spectrometer system (Thermo Fisher Scientific) equipped with a heated electrospray ionization source. The system was operated in positive- or negative-ion mode with the following optimized parameters: spray voltage, +3500 V or –2500V; vaporizer temperature, 400 °C; ion transfer tube temperature 375 °C; sheath gas flow rate, 75 arbitrary units (ARB); auxiliary gas flow rate, 25 Arb; sweep gas flow rate, 2 Arb; and collision gas pressure, 1.5 millitorr. The cycle time was 0.5 sec. Data analysis was performed using TraceFinder Software (Thermo Fisher Scientific).

## 2.5. Method Validation

The assay was validated for clinical testing according to the U.S. Food and Drug Administration (FDA) and the Clinical and Laboratory Standards Institute (CLSI) guidelines assessing the following performance characteristics: analytical measurement range (AMR)/linearity, sensitivity, precision, accuracy, matrix effect, specificity, and carryover [12–15]. The stability of the analytes at various temperatures was studied in serum and plasma.

### 2.5.1. Matrix effect

The equivalency between the calibrator matrices (RECIPE serum and Bio-Rad Lyphochek drug-free serum) and leftover patient serum (n = 6) or plasma (n = 6) samples was evaluated in a mixing study. Blank patient samples without AED analytes were mixed 1:1 with the calibrator matrix, either serum-based RECIPE Calibrator (Level 2) or PENTOB calibrator (Level 2). Patient serum or plasma samples, calibrator matrices, and their corresponding 1:1 mixtures were extracted and injected in an alternating sequence. The response ratio (peak area/IS area) of the blank sample and the candidate calibrator matrix were obtained. These were used to calculate a predicted (theoretical) response ratio as follows: ([calibrator matrix response ratio + patient sample response ratio]/2). The experimental response ratio was obtained from the 1:1 mixtures. For each analyte, the results from the six serum or plasma samples were averaged. Bias was calculated as [(experimental mixture response ratio – theoretical mixture response ratio) / theoretical mixture response ratio] × 100. A bias within ±20% was considered acceptable.

Potential matrix effects were evaluated qualitatively using the post-column infusion method [16]. The AED analytes were spiked into a buffer solution (2 mM ammonium acetate in 50% methanol) in two groups: Group 1, ZONIS, FELBA, LMTR, 10HYDROCARB, CARBEP, and PENTOB, and Group 2, PBALIN, GABA, ETHOS, LACOS, PRIM, RUFIN, TOPIR, BRIV, and PRMP. The constant infusion of neat mixtures of each group was accompanied by injections of blank (50:50 MPA:MPB), extracts from 10 blank serum patient samples, and extracts from 10 blank plasma patient samples. Selected reaction monitoring (SRM) transitions of the analytes were monitored during the entire run. The traces of the extracted samples were compared to the mobile phase blank to evaluate the degree of ion suppression or enhancement.

### 2.5.2. Sensitivity, linearity, and AMR

The target AMRs were established using a data-driven approach that incorporated therapeutic ranges and 2 years of historical patient results from the current laboratory method, whether in-house or send out. For the lower limit of the AMR, defined as the lower limit of quantitation (LLOQ), the therapeutic range was prioritized to ensure accurate measurement at clinically meaningful concentrations. For the upper limit, consideration was given to the frequency of high concentration results and the potential need for sample dilution, which can delay reporting. Therefore, we targeted an AMR upper limit that remained clinically appropriate and minimized the number of required dilutions. The LLOQ was evaluated in blank serum pools spiked at the target concentrations and analyzed in 20 replicates for at least 3 days. The assay linearity was assessed using blank serum pools spiked to the upper limit of quantitation (ULOQ) for each analyte and diluted serially to the LLOQ. Each level was extracted in triplicate. The data were analyzed using the linearity and calibration verification module of EP Evaluator (Data Innovations, Colchester, VT, United States).

### 2.5.3. Selectivity

Assay selectivity was assessed through interference studies [13,14]. Potential interference from commonly used drugs was tested using an Interference Mix Kit (Cerilliant) that includes seven vials of several drugs in methanol or acetonitrile solvent, Liquichek Immunoassay Plus Control Level 3 (BioRad), and Liquid Unassayed Multiqual Chemistry Control Level 3 (BioRad). The compounds in these materials and their concentrations are listed in Supplementary Table S2. The interference mixes were diluted 10-fold in Lyphochek drug-free serum to reflect concentrations potentially present in patient samples. The diluted interference mixes and undiluted controls were extracted and injected. Each material was evaluated for interference. “No interference” was defined as no analyte peak area > 20% of the LLOQ [13], except for the tested AED analytes that were already present in the commercial mixes and QC materials (Mix 2: PBALIN, GABA; Mix 3: LACOS, RUFIN; Mix 4: LMTR; Mix 5: ZONIS, TOPIR; Liquichek Immunoassay Plus Control Level 3: ETHOS, PRIM). For these analytes, we calculated the concentration recovery and defined acceptability as an 80–120% recovery from the expected concentration. Additionally, AMOB interference was investigated because AMOB is a structural isomer of PENTOB [16]. Solutions containing AMOB (40 ng/mL) and/or PENTOB (40 ng/mL) in 30% methanol were injected. Chromatograms of the three solutions were compared to assess chromatographic separation.

We evaluated the effect of icterus (I), lipemia (L), and hemolysis (H). In this experiment, patient hemolysates (H index, ~1000 mg/dL), commercial intralipid (target L index, ~1000 mg/dL), and icteric patient sample (I index, ~30 mg/dL) were individually mixed 1:1 with two patient sample pools containing all AED analytes at concentrations within the therapeutic range (Low) and close to the ULOQ (High). The neat samples and mixes were extracted in triplicate and injected in a randomized order. The observed response ratio of each mixture was compared to the predicted response ratio.

### 2.5.4. Precision and accuracy

The precision of each analyte was evaluated using RECIPE’s ClinChek® Antiepileptics 5 Serum Controls (Level 1 and Level 2) and a 1:1 mix of Level 1 and Level 2 for a total of three QC materials. Intraday precision was calculated from 10 replicates in a single analytical run. Interday precision was calculated from two replicates per day for 10 days. The acceptability criteria were CV < 10%.

At least 20 serum or plasma samples were measured in the assay being evaluated and in a previously validated method for clinical use in a Clinical Laboratory Improvement Amendments-certified laboratory. When necessary, samples were spiked to produce analyte concentrations covering the AMR. The results were analyzed using Deming regression and Bland-Altman plots (GraphPad Prism v9). The expected overall bias was ≤50% of total allowable error (TAE), and 95% of the samples were expected to have bias within the TAE (Supplementary Table S3).

### 2.5.5. Carryover

Sample carryover was evaluated using samples spiked with standard materials at concentrations twice the ULOQ (High, H) and near the LLOQ (Low, L). Replicates of the samples were extracted and injected as follows: L1, L2, L3, H1, H2, L4, H3, H4, L5, L6, L7, L8, H5, H6, L9, H7, H8, L10, H9, H10, L11. The EP Evaluator Carryover module was used for evaluation. To calculate carryover, the average low-low and high-low results were determined. A low-low is the concentration of a low sample that immediately follows a low sample, whereas a high-low is the concentration of a low sample that immediately follows a high sample. The carryover is then calculated as the mean of the high-low results minus the mean of the low-low results. The error limit is defined as the standard deviation of the low-low results multiplied by 3. The carryover experiment passes if the amount of carryover is less than the error limit.

### 2.5.6. Sample stability

The stability of the 15 AED analytes at different storage conditions was evaluated in serum and plasma spiked to low, mid, and high concentrations. The samples were stored at an ambient temperature (19.5 to 24 °C) for 8 h and 24 h, refrigerated (4 °C) for 7 and 14 days, and frozen (≤−20 °C) for 30 days and after two freeze-thaw cycles. The stability of extracted samples stored in the autosampler at 4 ± 2 °C was evaluated after 3 and 7 days. The samples were considered stable if the concentration bias was within ±20% of the baseline concentration.

## 3. Results

### 3.1. Method Development and Chromatographic Separation

The chemical structures of all drugs analyzed are shown in Figure 2. SRM transitions, ion mode, and optimized CE for each analyte are listed in Table 1. Most precursor ions are [M + H]^+^ or [M − H]^−^ in ESI mode, except for [M − CH_3_NO_2_]^+^ for FELBA due to the loss of carbamic acid, [M + NH_4_]^+^ for TOPIR with an ammonium adduct, and [M − CH_3_NO]^+^ for BRIV due to the loss of formamide [3,17]. While we aimed to use two product ions, one as the quantifier and another as the qualifier, this was not possible for ETHOS. Other authors reported using the precursor ion as quantifier or one ion transition (no qualifier) in ESI negative mode—that is, 140 > 140 m/z or 140 > 42 m/z [18,19]. We, too, had trouble finding two stable transitions and defined the method to monitor the precursor ion to quantify (140.1 > 140.1 m/z) and one fragment to qualify (140.1 > 42.0 m/z). The signal intensities for ETHOS and PENTOB were lower than desired at a 5 µL injection volume and resulted in suboptimal chromatography. This could be resolved either by reducing the dilution factor during the final step of the sample preparation or by increasing injection volume. To maintain a standardized sample preparation workflow, we chose to increase the injection volumes as optimized during method development to 10 µL for PENTOB and 20 µL for ETHOS.

**Figure 2.**
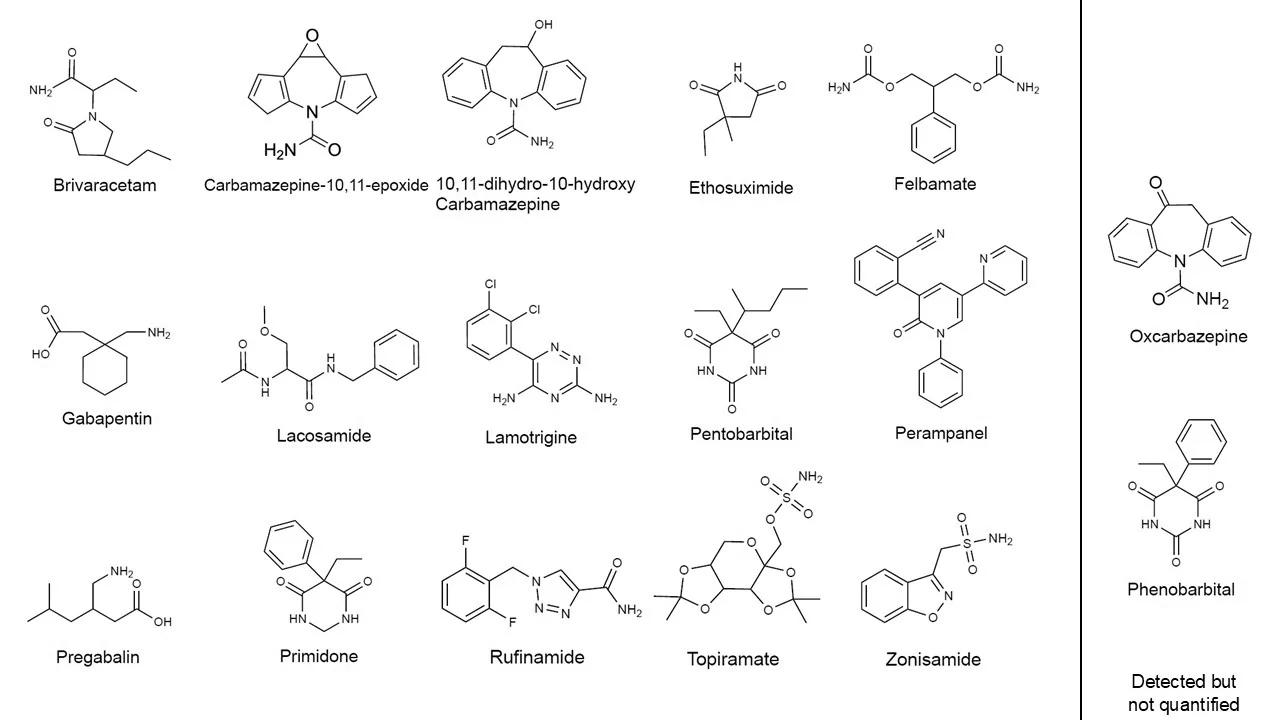
Chemical structures of AEDs.

**Table 1.** SRM transitions, polarity, RT, CE, and collection window for analytes and IS.

Analyte/IS	RT	Polarity	Collection window (min)	Precursor ion (m/z)	Quant/qual ions (m/z)	CE (V)
PBALIN	0.36	+	0.05–0.75	160.1	142.1 / 124.1	63
PBALIN-^13^C_3_				163.2	145.2 / 127.2	60
GABA	0.48	+	0.15–0.90	172.1	154.1 / 137.1	68
GABA-^13^C_3_				175.2	157.1 / 140.1	66
ETHOS	1.21	–	0.85–1.60	140.1	140.1 / 42.0	77
ETHOS-^2^H_3_				143.1	143.1 / 42.0	79
ZONIS	1.44	+	1.10–1.85	213.1	132.0 / 77.0	79
ZONIS-^13^C_6_				219.1	138.1 / 82.1	80
LACOS	1.86	+	1.50–2.25	251.2	108.1 / 91.1	66
LACOS-^13^C,^2^H_3_				255.2	108.1 / 91.1	67
RUFIN	1.98	+	1.60–2.35	239.1	127.0 / 222.0	75
RUFIN-^15^N,^2^H_2_				242.1	129.0 / 224.1	74
PRIM	2.02	+	1.65–2.40	219.1	162.1 / 119.0	77
PRIM-^2^H_5_				224.1	167.1 / 124.1	72
FELBA	2.13	+	1.75–2.50	178.1	117.0 / 115.0	73
FELBA-^2^H_4_				182.1	121.0 / 120.0	73
LMTR	2.32	+	1.95–2.70	256.0	211.0 / 145.0	161
LMTR-^13^C,^15^N_4_				261.1	214.0 / 145.0	133
PHENOB*	2.48	–	2.10–2.85	231.1	188.0 / 42.0	68
TOPIR	2.65	+	2.25–3.00	357.2	264.1 / 282.1	83
TOPIR-^2^H_12_				369.2	270.1 / 288.1	83
10HYDROCARB	2.72	+	2.35–3.10	255.2	194.1 / 192.2	66
10HYDROCARB-^3^C_6_				261.2	200.1 / 198.2	61
CARBEP	2.74	+	2.40–3.15	253.1	180.1 / 236.1	80
CARBEP-^13^C_6_				259.2	186.1 / 242.1	79
BRIV	2.95	+	2.55–3.30	168.1	55.0 / 140.1	88
BRIV-^2^H_3_				171.2	55.0 / 143.1	91
OXCARB*	2.99	+	2.65–3.35	253.1	236.0 / 180.1	90
PENTOB	3.5	–	3.15–3.90	225.1	182.1 / 42.0	87
PENTOB-^2^H_5_				230.1	187.1 / 42.0	87
PRMP**	3.96	+	3.60–4.22	350.1	219.1 / 247.1	161

* Not quantified in the method.

** Uses LACOS-^13^C,^2^H_3_ as IS.

R, retention time.

We achieved adequate chromatographic separation of the AEDs (Figure 3). The retention time of each AED analyte was relative to the beginning of the data collection window at 0.7 min. CARBEP and OXCARB are isomers, with shared precursor and product ions (253.1 > 180.1/236.0 m/z) [20,21]. They were chromatographically separated at a resolution of 0.24 min (Supplementary Figure S1). OXCARB was not quantified in our method or routinely because although it is pharmacologically active, it is rapidly metabolized to 10HYDROCARB.

**Figure 3.**
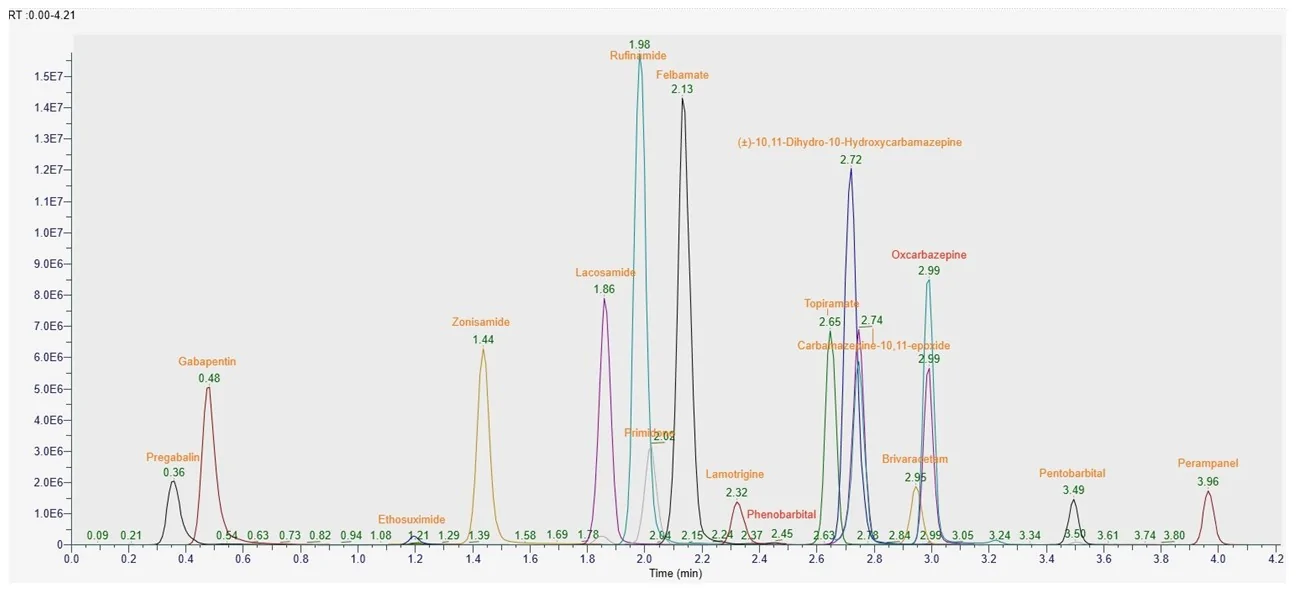
Chromatogram overlay of spiked AEDs detected by this method with their respective retention times. The retention time shown is relative to the beginning of data collection at 0.7 min.

### 3.2. Method Validation

#### 3.2.1. Matrix effect

We found matrix equivalency between serum/plasma and the calibrators used, based on the response ratios obtained from the 1:1 mix of the matrix and the calibrator (Supplementary Table S4). The response ratio was calculated as the peak area of the target analyte divided by the peak area of its corresponding isotopically labeled IS. The expected response ratio (derived from each neat sample) and the observed response ratios from the 1:1 mix were comparable, with a bias of less than 10%. No significant ion enhancement or ion suppression was observed in the post-column infusion qualitative matrix effect study for any of the analytes. For a simplified representation of the results, we show the graphs for PBALIN, RUFIN, 10HYDROCARB, and PRMP and the injection of four serum and plasma samples (Supplementary Figure S2). The remaining AEDs demonstrated similar performance (data not shown).

#### 3.2.2. Sensitivity, linearity, and AMR

The CV at the target LLOQs ranged from 2.3% to 15.9% for all analytes (Table 2). The analytes demonstrated linearity across the AMR, despite not all calibrators covering the full range of the AMR (e.g., PBALIN, ZONIS, LACOS, FELBA, LMTR, TOPIR, 10HYDROCARB, BRIV, and PRMP) (Table 2 and Supplementary Table S1). Linearity and the AMR were assessed by spiking each AED analyte into a blank patient serum pool at concentrations targeting the upper limit of the AMR, followed by serial dilutions near the LLOQ (Table 2). The percent recoveries across the measured range were between 80.0% and 118.3% (Table 2). The percent recovery was calculated by dividing the mean (n = 3) of the measured concentration at each level by the target concentration.

**Table 2.** Linearity, AMR, and LLOQ.

Analyte	Target AMR (µg/mL)	Verified range (µg/mL)	No. levels	Recovery (%)	Linear equation	CV (%) at LLOQ
PBALIN	0.5–20.0	0.3–21.2	6	93.5–108.8	y = 1.051 × −0.0045	9.0
GABA	0.5–30.0	0.6–32.0	7	108.0–111.0	y = 1.093 × −0.0052	6.6
ETHOS	10.0–150.0	10.3–150.0	5	99.6–109.5	y = 1.000 × −0.5100	3.7
ZONIS	2.5–100.0	1.8–101.5	7	99.9–117.7	y = 1.003 × +0.1768	2.3
LACOS	0.5–30.0	0.4–29.8	7	80.0–100.0	y = 1.002 × −0.0700	5.7
RUFIN	0.5–50.0	0.4–43.6	8	87.3–102.1	y = 0.888 × +0.1198	9.8
PRIM	0.5–30.0	1.7–27.3	5	91.1–92.7	y = 0.909 × +0.0397	5.1
FELBA	2.0–150.0	0.8–148.2	8	91.4–107.5	y = 1.029 × +0.0162	3.0
LMTR	0.5–30.0	0.5–33.6	7	96.6–112.0	y = 1.069× −0.1829	11.4
TOPIR	0.5–30.0	201.0–31.0	5	103.2–110.8	y = 1.048 × +0.2235	3.2
10HYDROCARB	0.5–100.0	0.3–101.3	9	81.8–101.5	y = 1.006 × −0.1327	15.9
CARBEP	0.5–10.0	0.4–11.6	6	108.0–115.6	y = 1.150 × −0.0533	6.7
BRIV	0.1–10.0	0.08–10.6	8	99.1–107.3	y = 1.039 × −0.0171	7.6
PENTOB	2.0–100.0	1.7–96.9	7	94.2–110.7	y = 0.953 × +0.0877	7.9
PRMP	0.02–5.00	0.02–4.60	9	85.7–118.3	y = 0.890 × +0.0015	14.8

AMR: Analytical measurement range; LLOQ: Lower limit of quantitation.

#### 3.2.3. Selectivity

We did not identify any exogenous interference for the AEDs from the components in the various commercial multicomponent solutions. In the presence of interference mixes, the AED peaks were below the LLOQ. When AEDs were known to be present in these solutions, recovery was within 15% of the expected concentrations. This method did not accomplish chromatographic separation of the isomers AMOB and PENTOB (Supplementary Figure S3) [22]. We did not observe interference from I, L, or H (Supplementary Table S5).

#### 3.2.4. Precision and accuracy

The assay demonstrated excellent intraday and interday precision, ranging from 0.9% to 6.5% and 2.2% to 7.3%, respectively (Table 3). The method accuracy was evaluated for the AED analytes using at least 20 samples, including residual serum or plasma specimens or spiked samples, compared to a previously validated method in our laboratory or a reference laboratory. The reference methods included HPLC-UV (ZONIS, FELBA, LMTR, 10HYDROCARB, CARBEP, and PENTOB), quantitative enzyme IA (GABA and ETHOS), and LC-MS/MS (PBALIN, LACOS, RUFIN, PRIM, TOPIR, BRIV, and PRMP). All the analytes demonstrated acceptable accuracy by Deming regression analysis and Bland-Altman plots (Figure 4 for PBALIN, GABA, ZONIS, LACOS, BRIV, and PRMP and Supplementary Figure S4 for the remaining analytes).

**Table 3.** Intraday and interday precision.

Analyte	Mean (µg/mL)			Intraday CV (%)			Interday CV (%)		
	QC1	QC2	QC3	QC1	QC2	QC3	QC1	QC2	QC3
PBALIN	1.84	3.12	4.49	2.3	2.0	2.3	3.2	3.9	3.7
GABA	4.84	8.05	11.41	2.0	2.5	1.4	2.7	3.3	2.5
ETHOS	21.75	35.64	49.97	0.9	2.5	2.4	2.9	4.2	3.8
ZONIS	8.64	14.25	19.83	1.0	1.4	1.2	2.2	2.2	2.4
LACOS	2.54	4.32	6.12	1.3	2.0	2.3	3.4	4.5	3.2
RUFIN	7.89	13.13	18.43	1.4	1.4	1.9	4.0	3.8	4.0
PRIM	4.84	8.53	12.06	1.8	1.8	2.3	5.3	3.5	5.0
FELBA	19.88	33.01	46.40	1.9	2.0	1.0	3.2	2.9	3.3
LMTR	3.96	6.70	8.91	6.5	6.4	5.9	5.9	6.5	7.3
TOPIR	3.70	6.08	8.54	2.0	1.7	3.9	4.9	4.6	4.2
**10HYDROCARB**	7.83	13.07	18.25	1.1	1.1	2.7	3.6	3.8	3.7
CARBEP	1.70	2.86	4.01	1.9	2.3	1.8	2.9	2.6	3.0
BRIV	0.82	1.40	1.98	1.9	3.2	3.3	4.8	6.5	4.6
PENTOB	29.26	-	49.86	2.5	-	1.2	3.9	-	3.1
PRMP	0.275	0.438	0.628	0.9	1.8	2.1	4.0	6.7	3.9

All analytes except PRMP matched isotopically labeled IS. Acquiring an isotope-labeled IS for PRMP, a Schedule III controlled substance by the Drug Enforcement Administration (DEA), for routine use in a clinical laboratory was both difficult and prohibitively expensive. TDM of PRMP is a recent practice, with only a few methods available in reference laboratories. Recently, de Grazia and colleagues used LACOS-^2^H_3_ to quantify PRMP [23]. Our results demonstrated that LACOS-^13^C,^2^H_3_ could be used for accurate quantification of PRMP, both relative to another LC-MS/MS method in a reference laboratory and to target PRMP concentrations in spiked samples (Figure 4; Supplementary Figure S5).

**Figure 4.**
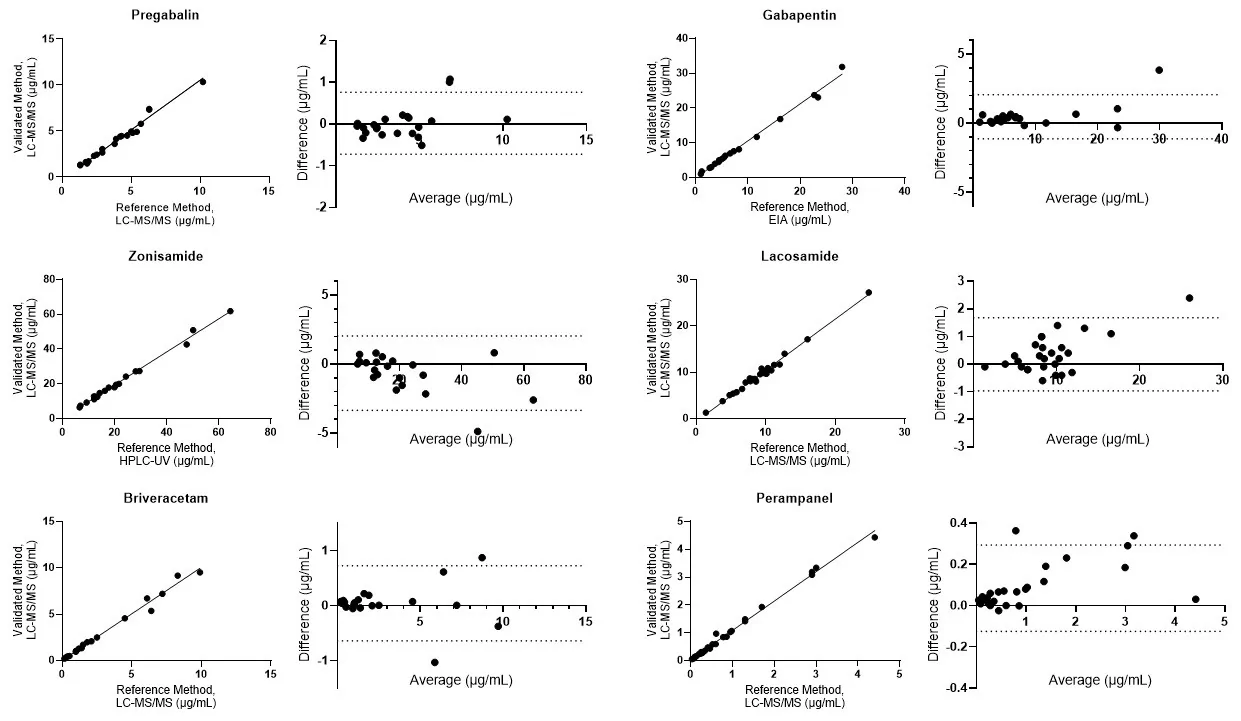
Accuracy by method comparison for representative analytes: PBALIN, GABA, ZONIS, LACOS, BRIV, and PRMP. Deming regression and Bland-Altman plots for the validated LC-MS/MS method and a comparative method. The dotted line in the Bland-Altman plots represents the 95% limits of agreement between the methods.

#### 3.2.5. Carryover

The method was not affected by carryover for any of the analytes, up to concentrations twice the upper limit of the AMR (Supplementary Table S6).

#### 3.2.6. Sample stability

Experiments were conducted to assess the stability of AEDs in serum and plasma across three clinically relevant concentrations. All AEDs were stable in serum and plasma after sample storage for 24 h at room temperature, 7 days refrigerated (4 °C), and 30 days frozen (−20 °C) and after two freeze-thaw cycles (Figure 5). Extracted samples were stable when stored in the autosampler for up to 7 days (Supplementary Figure S6). Only serum data are shown as plasma demonstrated comparable performance.

**Figure 5.**
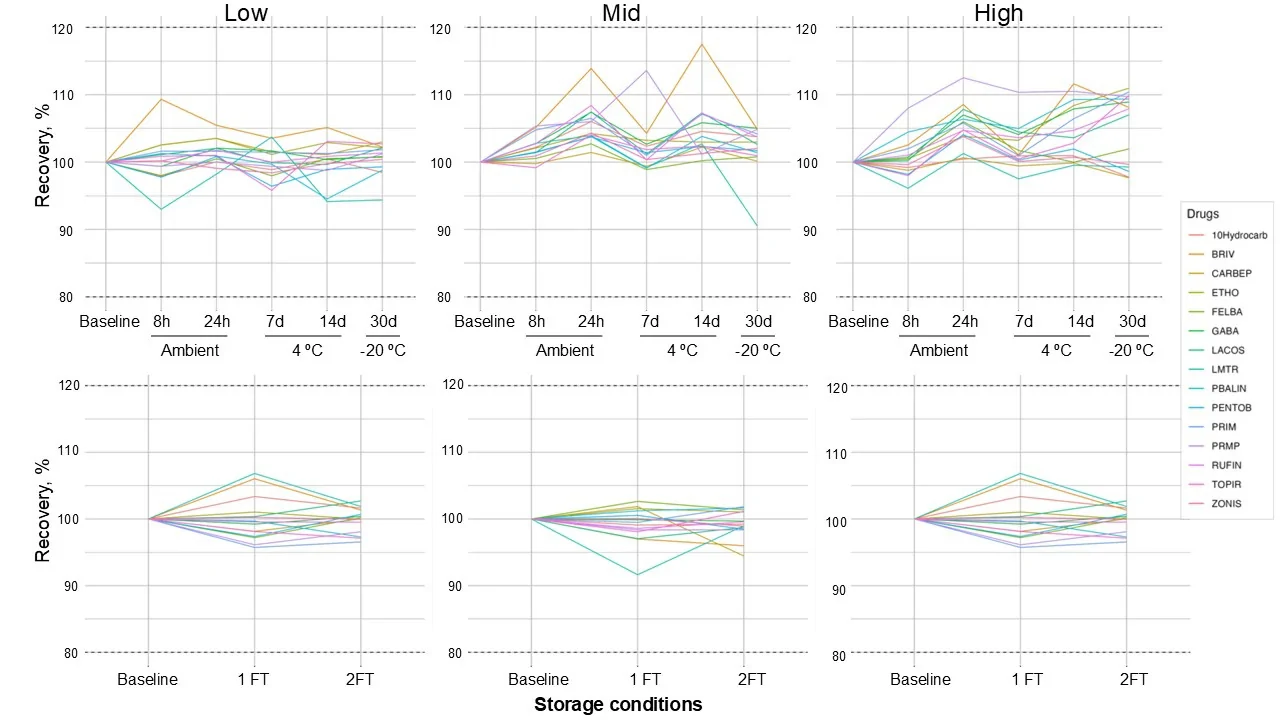
Effect of sample storage at various environmental conditions. *Top*: stability of serum at various storage conditions. *Bottom:* stability of serum after two freeze/thaw (FT) cycles.

## 4. Discussion

Here, we demonstrate a simple and rapid LC-MS/MS method to quantify multiple generations of AEDs for TDM applications. As illustrated in Figure 1, we leveraged a standardized sample preparation approach and method settings, along with a commercial calibrator set and an in-house calibrator for PENTOB. PENTOB, traditionally classified as a barbiturate with high abuse potential, is primarily used in a hospital setting as a sedative, as a preanesthetic, for emergency seizure control, and off-label to manage intracranial pressure. Given the limited scope of use, it is not surprising that it is excluded from the commercial calibrator set. However, due to its high toxicity, clinical practice supports monitoring PENTOB concentrations [22].

The method presented here has several advantages and practical improvements, complementing published approaches for quantifying AEDs by LC-MS/MS. Notably, it enables simultaneous quantification of a larger number of AEDs compared to other methods reported in the literature [24–27]. Similarly to our method, Shibata and colleagues developed an LC-MS/MS method capable of detecting 22 AEDs with a 10 min runtime; their method required three runs [3]. Importantly, their method did not use an IS; instead, they validated positional differences to investigate time-dependent changes of MS detection. Another group reported quantification of 16 AEDs using olanzapine as an IS [28]. However, these approaches are not desirable in contexts where isotopically labeled IS signal intensity is routinely monitored to assess signal suppression or enhancement, such as the monitoring required by the College of American Pathologists [29]. Additionally, best practice guidelines recommend the use of an IS for quantification, as this approach improves quality, accuracy, and precision; tracks chromatographic behavior; helps correct matrix effects; and enables quantification based on the ratio of the analyte to the IS in the calibrator [13]. Our method includes an analyte-matched IS for all compounds except PRMP due to its limited availability. This limitation may be addressed in the future as commercial options continue to expand.

A main feature of the method is the ease of use of a calibrator set, as preparing an in-house product containing all these analytes would be very challenging. For the calibrators in the set that do not cover the AMR—as defined by each laboratory—laboratories may need to verify the AMR at least every 6 months. In our experience, this was the case for some analytes (listed in Supplementary Table S1). Moreover, we acknowledge that supply chain issues could significantly disrupt our operations, and backup calibrator plans are needed. The method offers flexibility to test some or all analytes in the calibrator mix. For example, we opted not to validate PHENOB and continue using an IA method. However, PHENOB could be easily added if needed. Jiang and colleagues, for instance, reported the successful development of 24 AEDs using the commercial calibrator within a runtime of 5.2 min [30]. The applicability of their method is limited by the lack of performance characteristics data, preventing us from assessing analytical performance. Accuracy was assessed using QC or by comparison to IA for only four analytes, while matrix effects were not evaluated. This leaves questions about the overall performance of the assays for TDM in a clinical laboratory setting. This group successfully used analyte matched IS, although it relied on a commercial IS mix product, which could significantly increase the cost of implementing this method. A common challenge for multianalyte methods is the cost and effort associated with preparing an isotopically labeled IS mixture. To improve cost-efficiency, our method was subdivided. PENTOB-^2^H_5_ is prepared as a stand-alone solution, while the remaining AEDs are divided into two smaller groups. One IS mixture includes higher volume tests (~≥100 per month), including LMTR, 10HYDROCARB, ZONIS, LACOS, and TOPIR and a subset of low-volume tests (<15 per month), including PRMP, which shares IS with LACOS [23], CARBEP, and FELBA. The second IS mixture is used for midvolume tests (~30–50 per month), including GABA, PBALIN, ETHOS, RUFIN, BRIV, and PRIM.

We highlight the short separation time of this method and its ability to separate the isomers OXCARB and CARBEP. Unfortunately, the method lacks chromatographic separation between structural isomers AMOB and PENTOB. AMOB belongs to the barbiturate class and is a DEA Schedule II controlled substance. The tablet form, known as Amytal (Eli Lilly and Company, Indiannapolis, IN, US), was discontinued in the United States in the 1980s. The injectable form, Amytal sodium, was previously available for intravenous administration in highly restricted procedures [31,32] but has recently been discontinued [33]. Although AMOB and PENTOB can be separated chromatographically [34], we did not pursue further methods of development. The risk of a patient requiring TDM having both PENTOB and AMOB present simultaneously is low. Based on a laboratory’s risk assessment, a comment alerting personnel about this potential interference can be appended to the report.

Last, the implementation of this method added two new tests to our menu, PRMP and BRIV, and we have seen an increasing number of orders since their implementation. PRMP is a first-in-class, orally active, noncompetitive alpha-amino-3-hydroxy-5-methyl-4-isoxazolepropionic acid receptor antagonist. In the United States, PRMP (brand name Fycompa®) is indicated for partial-onset seizures, with or without secondarily generalized seizures, in patients aged 4 years and older and as adjunctive therapy for primary generalized tonic-clonic seizures for patients aged 12 years and older [35]. Several characteristics make it a strong candidate for TDM [36,37]: It is approximately 95% protein-bound, has a long half-life of about 105 h, and its clearance is markedly affected by drug-drug interactions, being increased by enzyme-inducing agents such as carbamazepine and phenytoin and inhibited by agents such as ketoconazole. Additionally, it has a narrow therapeutic range. CARBEP, phenytoin and OXCARB can significantly decrease PRMP concentrations through a CYP3A4 mediated pathway [35,36]. This supports the need to monitor PRMP concentrations in the blood as well as coadministered AEDs. Only a limited number of published methods are available for PRMP, either alone or in small panels, primarily utilizing HPLC and LC-MS/MS [23,38–40], and even fewer methods are reported for BRIV [41–43]. A recent review highlights the adverse effects of third-generation antiseizure medications such as PRMP and BRIV, which are among the five drugs approved for the treatment of focal onset seizures in clinical practice [44]. As the use of third-generation AEDs continues to expand, an increased reliance on TDM is anticipated to optimize their efficacy and mitigate the risk of preventable serious adverse effects.

## 5. Conclusion

This streamlined LC-MS/MS method offers several advantages for monitoring AEDs in a clinical laboratory. It enables accurate and efficient quantification across a broad panel of analytes, including the separation of clinically relevant isomers and the use of ion-ratio criteria to support quality assurance. The method is also flexible and cost-effective: It accommodates analytes not included in the commercial calibrator set, such as PENTOB, and allows the analytes to be divided into manageable groups for IS preparation based on test volume or common co-ordering patterns. Overall, this LC-MS/MS approach supports the routine analysis of 15 AEDs with a 7.3 min runtime, meeting both clinical and operational needs.

## Supplementary Material

Supplemental Material

## Data Availability

Data are available upon reasonable request, with appropriate restrictions. Data are not publicly available because they consist of de-identified clinical laboratory data generated under Institutional Review Board approval.

## Abbreviations

10HYDROCARB: (±)-10,11-dihydro-10-hydroxycarbamazepine
AED: Antiepileptic drugs
AMOB: Amobarbital
AMR: Analytical measurement range
ARB: Arbitrary units
BRIV: Brivaracetam
CARBEP: Carbamazepine-10,11 epoxide
CV: Coefficient of variation
DEA: Drug Enforcement Administration
ETHOS: Ethosuximide
FDA: Food and Drug Administration
FELBA: Felbamate
GABA: Gabapentin
H: Hemolysis
HPLC: High-pressure liquid chromatography
I: Icterus
IA: Immunoassay
IS: Internal standard
L: Lipemia
LACOS: Lacosamide
LLOQ: Lower limit of quantitation
LMTR: Lamotrigine
MPA: Mobile phase A
MPB: Mobile phase B
OXCARB: Oxcarbazepine
PBALIN: Pregabalin
PENTOB: Pentobarbital
PHENOB: Phenobarbital
PRIM: Primidone
PRMP: Perampanel
QC: Quality control
RUFIN: Rufinamide
TAE: Total allowable error
TAT: Turnaround time
TDM: Therapeutic drug monitoring
TOPIR: Topiramate
ULOQ: Upper limit of quantitation
ZONIS: Zonisamide

## Supplementary Material

Supplemental Material Available at https://doi.org/10.21627/hs00ns84
