# Supplementary material for: A Multiplex LC-MS/MS Approach for Quantifying Antiepileptic Drugs for Therapeutic Drug Monitoring: Validation of 15 Representative Tests
Source: J Mass Spectrom Adv Clin Lab. 2026 Aug 26;39(1):hs00ns84. doi: 10.21627/hs00ns84 (PMC13750511; doi:10.21627/hs00ns84)
Supplement: Supplementary file 1 [file jmsacl-39-1-hs00ns84-s001.docx]

**Supplemental Material for**

A Multiplex LC-MS/MS Approach for Quantifying Antiepileptic Drugs for Therapeutic Drug Monitoring: Validation of 15 Representative Tests

^‡^Xiaoying Tang^1^, ^‡^Ruhan Wei^1,2^, Emily Chegwidden^1^, Richard Giles^1^, Adam M. Kutnick^1^, Drew Payto^1^, Jessica M. Colón-Franco^*1^

^1^Department of Laboratory Medicine, Cleveland Clinic Foundation, Cleveland, OH, United States

^2^Department of Pathology, Duke University School of Medicine, Durham, NC, United States

^‡^These authors contributed equally.

**Supplementary Table S1.** Concentrations of calibrators in the ClinCal® Antiepileptics 5 Serum Calibrator (RECIPE, Munich, Germany)

| **Analyte** | **Level 1 (µg/mL)** | **Level 2 (µg/mL)** | **Level 3 (µg/mL)** | **Target AMR (µg/mL)** | **AMR covered by calibrators** |
| --- | --- | --- | --- | --- | --- |
| PBALIN | 0.671 | 3.8 | 11.5 | 0.5−20.0 | No |
| GABA | 1.79 | 8.91 | 27.6 | 0.5−30.0 | Yes |
| ETHOS | 8.27 | 39.8 | 121.0 | 10.0−150.0 | Yes |
| ZONIS | 2.93 | 14.3 | 42.9 | 2.5–100.0 | No |
| LACOS | 0.85 | 4.76 | 13.8 | 0.5−30.0 | No |
| RUFIN | 2.77 | 14.2 | 42.3 | 0.5−50.0 | Yes |
| PRIM | 1.63 | 9.56 | 29.1 | 0.5−30.0 | Yes |
| FELBA | 7.02 | 36.6 | 105.0 | 2.0−150.0 | No |
| LMTR | 1.34 | 7.18 | 20.4 | 0.5−30.0 | No |
| TOPIR | 1.20 | 5.83 | 18.1 | 0.5−30.0 | No |
| 10HYDROCARB | 2.67 | 13.8 | 42.5 | 0.5−100.0 | No |
| CARBEP | 0.585 | 3.14 | 8.47 | 0.5−10.0 | Yes |
| BRIV | 0.283 | 1.42 | 4.12 | 0.1−10.0 | No |
| PRMP | 0.0993 | 0.488 | 1.43 | 0.02−5.0 | No |

Calibrator-assigned concentrations are lot dependent. AMR coverage was defined as “yes” if Level 1 and 3 were close to the AMR limits (e.g., ~±TAE).

**Supplementary Table S2.** Analytes present in the interference materials.

| **Interference material** | **Compounds (concentration)** |
| --- | --- |
| Mix 1, Cerilliant | Acetaminophen (1 mg/mL), caffeine (1 mg/mL), (-)-cotinine (1 mg/mL), (-)-nicotine (1 mg/mL), ibuprofen (1 mg/mL), naproxen (1 mg/mL), phentermine (100 μg/mL), R,R(-)-pseudoephedrine (100 μg/mL) |
| Mix 2, Cerilliant | Gabapentin (100 μg/mL), pregabalin (100 μg/mL), valproic acid (100 μg/mL), vigabatrin (100 μg/mL) |
| Mix 3, Cerilliant | Aripiprazole (100 μg/mL), lacosamide (100 μg/mL), oxcarbazepine (100 μg/mL), (+)-propoxyphene (50 μg/mL), rufinamide (100 μg/mL), warfarin (1 mg/mL) |
| Mix 4, Cerilliant | Alprazolam (100 μg/mL), cimetidine (100 μg/mL), citalopram HBr (100 μg/mL), clonazepam (100 μg/mL), clopidogrel bisulfate (100 μg/mL), dextromethorphan (50 μg/mL), fluconazole (100 μg/mL), hydrochlorothiazide (100 μg/mL), lamotrigine (100 μg/mL), methylphenidate HCl (100 μg/mL), omeprazole (100 μg/mL), L-thyroxine (5 μg/mL) |
| Mix 5, Cerilliant | Carbamazepine (100 μg/mL), levetiracetam (100 μg/mL), metformin HCl (100 μg/mL), phenobarbital (100 μg/mL), R(-)-phenylephrine HCl (100 μg/mL), phenytoin (100 μg/mL), sertraline HCl (100 μg/mL), topiramate (100 μg/mL), zolpidem tartrate (100 μg/mL) |
| Mix 6, Cerilliant | Amlodipine besylate (5 μg/mL), atorvastatin calcium salt (100 μg/mL), azithromycin dihydrate (100 μg/mL), bupivacaine HCL ∙ H2O (100 μg/mL), cetirizine (HCl)2 (50 μg/mL), dimenhydrinate (100 μg/mL), lisinopril dihydrate (100 μg/mL), loratadine (100 μg/mL) |
| Mix 7, Cerilliant | Montelukast sodium salt (100 μg/mL), pioglitazone HCl (100 μg/mL), prednisolone (100 μg/mL), prednisone (50 μg/mL), procainamide HCl (100 μg/mL), simvastatin (100 μg/mL), zonisamide (100 μg/mL) |
| Liquichek Immunoassay Plus Control Level 3, BioRad^1^ | Acetaminophen (108 µg/mL), carbamazepine (14.1 µg/mL), digoxin (3.28 µg/mL), ethosuximide (130 µg/mL), gentamicin (8.35 µg/mL), immunoglobulin E (318 IU/mL), phenobarbital (51.3 µg/mL), phenytoin (22.7 µg/mL), primidone (12.6 µg/mL), quinidine (4.45 µg/mL), salicylate (38.9 mg/mL), theophylline (6.12 µg/mL), tobramycin (123 µg/mL), valproic acid (284 µg/mL) |
| Liquid Unassayed Multiqual Level 3, BioRad^1^ | Acetaminophen (150 µg/mL), acid phosphatase (total) (7 U/L), albumin (4.2 g/dL), alkaline phosphatase (300 U/L), alpha 1-antitrypsin (125 mg/dL), ALT/SGPT (200 U/L), amylase (325 U/L), amylase (pancreatic) (250 U/L), AST/SGOT (250 U/L), bilirubin (direct) (2 mg/dL), bilirubin (neonatal) (6.5 mg/dL), bilirubin (total) (7 mg/dL), calcium (13 mg/dL), carbamazepine (13 µg/mL), carbon dioxide (CO_2_) (26 mEq/L), chloride (120 mEq/L), cholesterol (HDL) (92.5 mg/dL), cholesterol (total) (275 mg/dL), copper (105 µg/dL), cortisol (40 µg/dL), creatine kinase (CK) (580 U/L), creatinine (7 mg/dL), digoxin (3 ng/mL), ethanol (200 mg/dL), gamma glutamyltransferase (GGT) (165 U/L), gentamicin (9.5 µg/mL), glucose (360 mg/dL), iron (225 µg/dL), iron (TIBC) (390 µg/dL), lactate (54 mg/dL), lactate dehydrogenase (350 U/L), LAP arylamidase (1.9 U/mL), lipase (130 U/L), lithium (2.3 mEq/L), magnesium (4 mg/dL), osmolality (539 mOsm/kg), phenobarbital (60 µg/mL), phenytoin (20 µg/mL), phosphorus (7 mg/dL), potassium (7.5 mEq/L), total protein (7 g/dL), salicylate (18 mg/dL), sodium (157 mEq/L), total T3 (2.5 ng/mL), total T4 (12.5 µg/dL), theophylline (25 µg/mL), thyroid stimulating hormone (15 µIU/mL), tobramycin (2 µg/mL), triglycerides (200 mg/dL), urea nitrogen (BUN) (70 mg/dL), uric acid (10 mg/dL), valproic acid (100 µg/mL), zinc (300 µg/dL) |

^1^Analyte concentrations are lot dependent.

**Supplementary Table S3.** Acceptability criteria used as percentage or absolute concentrations.

| **Test** | **TAE** | | **Precision** | | **Bias** | |
| --- | --- | --- | --- | --- | --- | --- |
|  | **%** | **µg/mL** | **%** | **µg/mL** | **%** | **µg/mL** |
| PBALIN | 20 | 1.0 | 10 | 0.5 | 10 | 0.5 |
| GABA | 20 | 1.0 | 10 | 0.5 | 10 | 0.5 |
| ETHOS | 20 | 5.0 | 10 | 2.5 | 10 | 2.5 |
| ZONIS | 20 | 2.0 | 10 | 1.0 | 10 | 1.0 |
| LACOS | 20 | 1.0 | 10 | 0.5 | 10 | 0.5 |
| RUFIN | 20 | 1.0 | 10 | 0.5 | 10 | 0.5 |
| PRIM | 20 | 1.0 | 10 | 0.5 | 10 | 0.5 |
| FELBA | 20 | 2.0 | 10 | 1.0 | 10 | 1.0 |
| LMTR | 20 | 1.0 | 10 | 0.5 | 10 | 0.5 |
| TOPIR | 20 | 1.0 | 10 | 0.5 | 10 | 0.5 |
| 10HYDROCARB | 20 | 1.0 | 10 | 0.5 | 10 | 0.5 |
| CARBEP | 20 | 1.0 | 10 | 0.5 | 10 | 0.5 |
| BRIV | 20 | 0.1 | 10 | 0.05 | 10 | 0.05 |
| PRMP | 20 | 0.02 | 10 | 0.01 | 10 | 0.01 |
| PENTOB | 20 | 2.0 | 10 | 1.0 | 10 | 1.0 |

**−Supplementary Table S4.** Results of the mixing study for matrix equivalency.

| **Analyte** | **Response ratios** | | | | | |
| --- | --- | --- | --- | --- | --- | --- |
|  | **Blank serum (n = 6)** | | | **Blank plasma (n = 6)** | | |
|  | **Predicted** | **Measured** | **% Bias** | **Predicted** | **Measured** | **% Bias** |
| **PBALIN** | 1.563 | 1.481 | −5.25% | 1.565 | 1.521 | −2.81% |
| **GABA** | 4.662 | 4.527 | −2.90% | 4.068 | 3.987 | −1.99% |
| **ETHOS** | 16.173 | 16.594 | 2.60% | 15.717 | 16.426 | 4.51% |
| **ZONIS** | 2.433 | 2.386 | −1.93% | 2.463 | 2.379 | −3.41% |
| **LACOS** | 2.096 | 2.011 | −4.06% | 2.109 | 2.087 | −1.04% |
| **RUFIN** | 8.759 | 8.796 | 0.42% | 8.939 | 8.844 | −1.06% |
| **PRIM** | 2.555 | 2.368 | −7.32% | 2.615 | 2.385 | −8.80% |
| **FELBA** | 25.231 | 25.245 | 0.06% | 25.673 | 25.534 | −0.54% |
| **LMTR** | 9.131 | 8.648 | −5.29% | 8.595 | 8.604 | 0.10% |
| **TOPIR** | 1.805 | 1.834 | 1.61% | 1.758 | 1.735 | −1.31% |
| **10HYDROCARB** | 6.043 | 5.986 | −0.94% | 6.050 | 5.924 | −2.08% |
| **CARBEP** | 0.893 | 0.872 | −2.35% | 0.896 | 0.864 | −3.57% |
| **BRIV** | 2.451 | 2.446 | −0.20% | 2.538 | 2.388 | −5.91% |
| **PRMP** | 0.233 | 0.219 | −6.01% | 0.236 | 0.229 | −2.97% |
| **PENTOB** | 0.392 | 0.381 | −2.81% | 0.389 | 0.377 | −3.08% |

**Supplementary Table S5.** Effect of lipemia, hemolysis, and icterus.

| **Analyte** | **Low pool (% Bias)** | | | **High pool (% Bias)** | | |
| --- | --- | --- | --- | --- | --- | --- |
|  | **Lipemia** | **Hemolysis** | **Icterus** | **Lipemia** | **Hemolysis** | **Icterus** |
| **PBALIN** | −5.0 | −1.5 | −2.8 | −3.0 | 0.4 | −2.6 |
| **GABA** | −3.8 | 0.1 | −0.2 | −6.8 | −4.8 | −4.6 |
| **ETHOS** | −1.1 | 3.1 | −1.9 | −1.2 | −1.0 | −0.5 |
| **ZONIS** | −2.4 | −4.0 | −5.3 | −3.1 | −2.2 | 0.4 |
| **LACOS** | −1.3 | 1.4 | 0.2 | −1.5 | 1.5 | 1.4 |
| **RUFIN** | −1.8 | 0.3 | −4.1 | −1.9 | 1.3 | −0.2 |
| **PRIM** | −9.9 | −7.3 | −11.2 | −11.7 | −11.6 | −11.7 |
| **FELBA** | 3.4 | −0.1 | −0.1 | −0.6 | 1.8 | 1.4 |
| **LMTR** | 3.9 | 4.6 | −7.3 | −5.9 | −11.2 | −10.9 |
| **TOPIR** | 0.3 | −1.7 | 2.8 | 1.1 | 7.1 | 7.0 |
| **10HYDROCARB** | 0.6 | −2.7 | −3.6 | −1.9 | −1.2 | 7.6 |
| **CARBEP** | −3.9 | −5.5 | −5.5 | −1.4 | −2.3 | 0.1 |
| **BRIV** | −8.0 | −5.7 | −4.0 | −1.4 | −2.1 | −1.7 |
| **PENTOB** | −5.9 | −2.0 | −4.9 | −3.7 | −5.4 | −4.3 |
| **PRMP** | −6.8 | −3.2 | −2.6 | −10.0 | −3.8 | −2.9 |

**Supplementary Table S6.** Carryover study.

| **Analyte** | **Low-low results^1^** | | **High-low results^2^** | | **Carryover^3^ (µg/mL)** | **Error limit^4^ (µg/mL)** |
| --- | --- | --- | --- | --- | --- | --- |
|  | **Mean (µg/mL)** | **SD** | **Mean (µg/mL)** | **SD** |  |  |
| **PBALIN** | 1.104 | 0.136 | 1.236 | 0.175 | 0.132 | 0.408 |
| **GABA** | 1.598 | 0.273 | 1.804 | 0.280 | 0.206 | 0.819 |
| **ETHOS** | 13.520 | 0.130 | 13.000 | 0.780 | −0.520 | 0.390 |
| **ZONIS** | 2.807 | 0.084 | 2.850 | 0.049 | 0.044 | 0.253 |
| **LACOS** | 1.680 | 0.200 | 1.840 | 0.260 | 0.160 | 0.600 |
| **RUFIN** | 1.632 | 0.269 | 1.862 | 0.372 | 0.230 | 0.807 |
| **PRIM** | 2.956 | 0.286 | 3.164 | 0.253 | 0.208 | 0.858 |
| **FELBA** | 1.755 | 0.095 | 1.829 | 0.043 | 0.074 | 0.284 |
| **LMTR** | 0.571 | 0.040 | 0.575 | 0.044 | 0.004 | 0.120 |
| **TOPIR** | 2.970 | 0.313 | 3.082 | 0.271 | 0.112 | 0.939 |
| **10HYDROCARB** | 0.484 | 0.054 | 0.550 | 0.038 | 0.066 | 0.161 |
| **CARBEP** | 0.677 | 0.012 | 0.683 | 0.017 | 0.007 | 0.036 |
| **BRIV** | 0.316 | 0.041 | 0.360 | 0.063 | 0.044 | 0.123 |
| **PENTOB** | 2.286 | 0.118 | 2.297 | 0.337 | 0.011 | 0.355 |
| **PRMP** | 0.045 | 0.003 | 0.048 | 0.003 | 0.003 | 0.008 |

To determine carryover, the average low-low and high-low results are calculated.

^1^Low-low results: concentration of a low sample that follows a low sample.

^2^High-low results: concentration of a low sample that follows a high sample.

^3^Carryover: mean of the high-low results minus the mean of the low-low results.

^4^Error limit: standard deviation of the low-low results multiplied by 3. The carryover is considered acceptable if the amount of carryover is below the error limit.

SD, standard deviation.

**Supplementary Figure S1.** Chromatographic separation of (**A**) carbamazepine-10,11-epoxide (CARBEP, 2.74 min), (**B**) CARBEP-13C_6_, and (**C**) oxcarbazepine (OXCARB, 2.98 min).

**Supplementary Figure S2.** Post-column infusion to evaluate ion suppression or enhancement for pregabalin, rufinamide, 10HYDROXYCARB, and perampanel.

Mobile phase blank (*black*) and four blank serum and plasma patient specimens: F-1 (*red*), F-2 (*green*), M-1 (*blue*), M-2 (*yellow*). *Pink:* chromatograph of AEDs.

**Supplementary Figure S3.** Chromatogram overlay of separate injections of solutions containing (**A**) AMOB, (**B**) PENTOB and PENTOB-2H_5_, and (**C**) AMOB, PENTOB, and PENTOB-2H_5_ with chemical structures inset.

*Black:* PENTOB SRM, *red:* PENTOB-2H_5_ SRM, *green:* AMOB SRM.

**Supplementary Figure S4.** Accuracy by method comparison for representative analytes: ethosuximide, rufinamide, primidone, felbamate, lamotrigine, topiramate, 10hydroxycarb, carbamazepine-10,11-epoxide, and pentobarbital. Deming regression and Bland-Altman plots for the validated LC-MS/MS method and a comparative method. The dotted line in the Bland-Altman plots represents the 95% limits of agreement between the methods.

**Supplementary Figure S5.** Correlation of perampanel by LC-MS/MS to target values (**A**) Deming regression and (**B**) Bland-Altman plots for the validated LC-MS/MS method and target values in the spiked samples. The dotted line in the Bland-Altman plots represents the 95% limits of agreement between the methods.

**Supplementary Figure S6.** Effect of refrigerated storage post sample extraction.
